# Efficacy of intravitreal ranibizumab combined with Ahmed glaucoma valve implantation for the treatment of neovascular glaucoma

**DOI:** 10.1186/s12886-016-0183-7

**Published:** 2016-01-09

**Authors:** Min Tang, Yang Fu, Ying Wang, Zhi Zheng, Ying Fan, Xiaodong Sun, Xun Xu

**Affiliations:** Department of Ophthalmology, Shanghai General Hospital of Nanjing Medical University, No.100 Haining Road, Hongkou District, Shanghai, 200080 China; Department of Ophthalmology, Shanghai General Hospital, Shanghai Jiao Tong University, School of Medicine, Shanghai, 200080 China

**Keywords:** Ahmed glaucoma valve, Ranibizumab, Neovascular glaucoma

## Abstract

**Background:**

Neovascular glaucoma is a refractive glaucoma. Recently, anti-VEGF factors have been used alone or in combination for the treatment of neovascular glaucoma. However, the medium- and long-term efficacy of such drugs remains to be evaluated. This study was to determine the efficacy of intravitreal ranibizumab combined with Ahmed glaucoma valve implantation for the treatment of neovascular glaucoma.

**Methods:**

In this prospective non-randomized study, 43 neovascular glaucoma patients (43 eyes) were assigned to receive either 0.5 mg intravitreal ranibizumab for three to 14 days before Ahmed glaucoma valve implantation (injection group, *n* = 21) or Ahmed glaucoma valve implantation alone (control group, *n* = 22). The patients were followed up for six to 12 months. Differences in surgical success rate, intraocular pressure, best corrected visual acuity, anti-glaucoma medications and postoperative complications were compared between the two groups. Surgical success was defined as IOP > = 6 mm Hg and < = 21 mm Hg, with or without the use of anti-glaucoma medications, and without severe complications or reoperation.

**Results:**

Of the 43 patients, 40 completed the 6-month follow-up and 37 completed the 1-year follow-up. Success rate was 73.7 % vs. 71.4 % at six months and 72.2 % vs. 68.4 % at 12 months in the injection group and the control group respectively. No significant difference was noted between the two groups (six months: *P* = 0.87, 12 months: *P* = 1.00). There were no significant differences in the two groups with respect to intraocular pressure, best corrected visual acuity, anti-glaucoma medications or postoperative complications at six months or 12 months.

**Conclusions:**

Single intravitreal ranibizumab (0.5 mg) before surgery has no significant effect on the medium- or long-term outcomes of neovascular glaucoma treated with Ahmed glaucoma valve implantation.

**Trial registration:**

Chinese Clinical Trial Registry (ChiCTR-OOC-14005709, Trial registration date: 2014-12-01)

## Background

Neovascular glaucoma (NVG) is a medical condition in which neovascularization involving the iris and the anterior chamber angle is accompanied by the formation of a fibrovascular membrane that results in secondary angle closure and obstructs the aqueous outflow. The main causes include diabetic retinopathy (DR), retinal vein occlusion (RVO), retinal artery occlusion (RAO) and ocular ischemic syndrome [1]. Ahmed glaucoma valve (AGV) implantation is an effective treatment for NVG, but the procedure is associated with poor outcomes [2]. A study found that vascular endothelial growth factor (VEGF) is a key factor causing NVG, as demonstrated by significantly higher VEGF levels in the aqueous humor of NVG patients [3]. VEGF levels in the aqueous humor are known to play a significant role in determining the outcomes of NVG patients after AGV implantation [4].

Because of their role in inhibiting intraocular neovascularization and mitigating damage to the blood ocular barrier due to leakage from new vessels, anti-VEGF factors have been used alone or in combination for the treatment of NVG. However, currently available evidence remains insufficient to confirm the effectiveness of such drugs. Ranibizumab (Lucentis) is now used in the treatment of age-related macular degeneration and macular edema as an anti-VEGF factor [5, 6], but it remains unclear whether ranibizumab will affect the efficacy of AGV implantation for NVG patients.

This prospective study was designed to compare the difference in efficacy at a follow-up of six to 12 months in NVG patients with or without a single intravitreal injection of ranibizumab (IVR) before AGV implantation.

## Methods

This study was a prospective, non-randomized, open-label, controlled study. This study enrolled patients admitted to the Department of Ophthalmology of Shanghai General Hospital from December 2012 to March 2014. Inclusion criteria were: 1) NVG patients (NVG was diagnosed by the presence of active neovascularization in the iris and/or angle, high intraocular pressure (IOP > 21 mm Hg, 1 mm Hg = 0.133 kPa, Goldmann applanation tonometer) and underlying ischemic retinal diseases); 2) IOP > 21 mm Hg, with or without anti-glaucoma medications or panretinal photocoagulation (PRP) before; 3) 18 to 85 years old; 4) patients who chose IVR before AGV implantation or AGV implantation only should complete a follow-up of six to 12 months. Exclusion criteria were: 1) patients combined with other types of glaucoma or other serious eye diseases; 2) patients who had received glaucoma surgery or other intraocular surgery in either eye; 3) patients who had received intravitreal injection in either eye within three months before surgery; 4) patients who failed to complete the scheduled follow-ups for various reasons; 5) IOP measurements were made inaccurate for various reasons; 6) IOP decreased (<= 21 mm Hg) after IVR and/or PRP; 7) cataract surgery or vitreous surgery was needed during the primary surgery; 8) surgery or intravitreal injection was required for both eyes; and 9) pregnant patients or patients combined with other serious uncontrolled medical diseases. This study was approved by the Ethics Committee of Shanghai General Hospital (registration number: 2012 K061), and it was registered with the Chinese Clinical Trial Registry (registration number: ChiCTR-OOC-14005709). All patients signed an informed consent before participation in this study.

Grouping method: NVG patients through preliminary screening would be educated about IVR on its effect, side-effect, risks, price and so on, then they chose to accept IVR before AGV implantation (injection group) or AGV implantation only (control group) at the discretion of themselves and signed an informed consent.

Endpoints were: 1) completion of the scheduled follow-up over the 6-month or 12-month period; 2) failure to be followed up as scheduled, being lost to follow up, undergoing intraocular surgery including cyclophotocoagulation during the follow-up period, or receiving intravitreal injection during the follow-up period (collectively referred to as dropouts).

We tried to do PRP for patients before IVR or AGV implantation if possible, and evaluated again whether they needed IVR or AGV implantation. As to those who could not accept PRP due to very high IOP or corneal edema pre-surgery, we applied this therapy just after surgery (usually 1 or 2 weeks later). Patients would not be enrolled if their IOPs were controlled by PRP before AGV implantation (IOP < = 21 mm Hg).

IVR was performed three to 14 days prior to AGV implantation. Under topical anesthesia, a needle was introduced through the conjunctival surface 3.8 mm from the corneal limbus in the affected eye for intravitreal injection of 0.5 mg/0.05 mL ranibizumab (Lucentis, 10 mg/mL; Novartis, Basel, Switzerland). The puncture site was pressed with a cotton swab for 5 to 10 s after the needle was withdrawn. IOP and light perception were examined. Sometimes anterior chamber paracentesis was performed in patients with higher IOP. All patients were observed more than three days. After that, AGV implantation would be performed, once the IOPs of patients reached 40 mm Hg during time, or it would be done two weeks after IVR. Otherwise, Patients would not be enrolled by the study if their IOPs were controlled just by IVR (IOP < = 21 mm Hg).

AGV implantation was performed under peribulbar anesthesia, a fornix-based conjunctival flap superior and temporal to the affected eye was prepared until the equator and a mitomycin C-soaked (0.4 mg/mL) cotton swab was applied to the area for two to five minutes before rinsing thoroughly with saline. An AGV (Model FP7) drainage plate was fixed with 6–0 suture on the surface of the sclera, with its anterior border 8 to 10 mm from the limbus. A 27G needle was introduced into the interlamellar space of the sclera 5 mm behind the limbus and pushed forward until into the anterior chamber, where viscoelastic agent was injected while the needle was being withdrawn slowly. A drainage tube was implanted into the anterior chamber two to three mm deep through the needle tract, and was mildly ligated with 8–0 absorbable suture (6 to 7 mm behind the limbus) before the conjunctival flap was tightly stitched. A small amount of aqueous humor was drained through a clear corneal if necessary incision to bring intraocular pressure slightly higher than normal IOP. All the procedures were completed by the authors.

Levofloxacin eye drops were applied at short intervals before surgery. Tobramycin and dexamethasone ophthalmic solution was applied postoperatively once every two hours for a total of three days, followed by four times a day for two weeks. Tropicamide or atropine was administered for two weeks for pupil dilation treatment where appropriate. Some patients received panretinal photocoagulation within one month after surgery. Anti-glaucoma medications were administered in light of IOP during follow-up.

The mean of three consecutive outpatient IOP measurements just before surgery was used as the baseline IOP. Patients were followed up on schedule (1–3d, 2w ± 1d, 1m ± 3d, 3m ± 5d, 6m ± 7d, 12m ± 14d) after surgery. Best corrected visual acuity (BCVA) and IOP were determined and slit-lamp microscopy with a preset lens was performed as a routine. Other tests including gonioscopy, ultrasound biomicroscopy, perimetry and retinal nerve fiber layer scan were conducted in selected patients where appropriate. IOP, surgical success rate, BCVA, anti-glaucoma medications and postoperative complications were used as major outcome measures at each follow-up time interval. Surgical success was defined as IOP > = 6 mm Hg and < = 21 mm Hg, with or without the use of anti-glaucoma medications, and without severe complications or reoperation [7]. Surgical failure was defined as IOP persistently < 6 mm Hg or > 21 mm Hg for more than two weeks, or loss of light perception, or the occurrence of any serious complication including endophthalmitis, corneal decompensation, malignant glaucoma, severe choroidal detachment (>180°), severe choroidal hemorrhage (>180°), retinal detachment, ocular atrophy, or displacement, withdrawal or exposure of drainage tube, or necessity of reoperation for other reasons.

Statistical analysis was performed using statistical package SAS9.13. Differences in gender and diagnoses at baseline and postoperative complications, and dropout rates during follow-up were compared between the two groups using chi-square, corrected chi-square test or Fisher’s exact probability test. Differences in age, IOP, BCVA, and medications at baseline and IOP decline, BCVA, and medications during follow-up were compared using the *t* test. Difference in success rates throughout follow-up was compared using the Log-Rank test. *P* value < 0.05 was considered statistically significant.

## Results

A total of 43 patients (43 eyes) were enrolled. The patients were divided into the injection group (*n* = 21), who received AGV implantation three to 14 days (average 8.6 ± 2.2 days) subsequent to IVR, and the control group (*n* = 22), who received AGV implantation alone. The baseline information of the two groups is presented in Table 1. There were no significant differences in baseline measures between the two groups.

**Table 1:** Characteristics of patients with NVG

	Injection Group	Control Group	*P*
Total Patients	21	22	
Gender			0.45
Male	10	13	
Female	11	9	
Age	60.1 ± 13.8 (32–81)	58.6 ± 17.3 (28–81)	0.76
Diagnosis			0.90
CRVO	8	7	
BRVO	2	2	
DR	11	13	
Baseline IOP (mm Hg)	46.4 ± 13.3 (24.5–76.0)	45.0 ± 14.9 (23.5–78.5)	0.74
Prior intravitreal injection	0	0	
NVI/NVA Degree			0.90
NVI only	3	2	
NVI&NVA (Open-angle)	5	4	
NVI&NVA (partial Closed-angle)	2	3	
NVI&NVA (Closed-angle)	11	13	
PRP before			0.55
none	12	9	
Incomplete	6	8	
complete	3	5	
BCVA (LogMAR)	1.1 ± 0.4 (0.3–1.6)	1.2 ± 0.4 (0.4–1.6)	0.65
Pre-medications	2.5 ± 0.5 (2–3)	2.6 ± 0.5 (2–3)	0.66

*Note*: The difference in gender was compared between the two groups using the chi-square test, the difference in NVI/NVA degree was compared using two-tailed Fisher’s exact test, and the differences in diagnosis and PRP before were compared using the corrected chi-square test. Differences in age, IOP, BCVA and anti-glaucoma medications were compared using the *t* test

Of the 43 patients, 40 completed the 6-month follow-up and 37 completed the 1-year follow-up. In particular, in the injection group two patients received intraocular surgery (at three months and six months) and one patient was lost to follow up (at 12 months). In the control group two patients received intraocular surgery (at six months and 12 months) and one patient were lost to follow up (at 12 months). The dropout rates were not significantly different between the two groups (six months: *P* = 0.52; 12 months: *P* = 0.95).

**Fig. 1:**
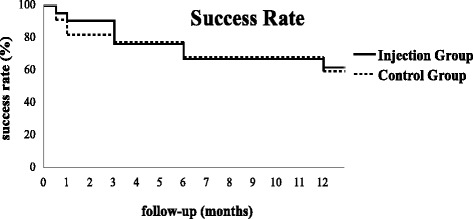
Success rates in both groups. Note: Difference in success rate throughout follow-up was compared between the two groups using the Log-Rank test (*P* = 0.84)

The mean IOPs at various time points throughout follow-up decreased significantly from baseline in both groups (ANOVA, α = 0.05). IOP rose gradually in both groups with the passage of time. IOPs at various time points throughout follow-up were not significantly different between the two groups (Table 2). The success rates declined gradually in both groups with the passage of time. Success rates at various time points throughout follow-up (Table 3) were not significantly different between the two groups (*P* = 0.84, Fig. 1).

**Table 2:** IOP (mm Hg) in both groups

	Pre-surgery	2 weeks	1 month	3 months	6 months	12 months
Injection Group IOP (mean ± SD)	46.4 ± 13.3	14.5 ± 4.4	16.9 ± 4.2	18.1 ± 3.8	20.5 ± 4.5	21.1 ± 4.2
(Minimum - Maximum)	(24.5–76.0)	(6.5–24.0)	(9.0–26.0)	(10.0–24.0)	(14.0–28.0)	(15.5–28.0)
Control Group IOP (mean ± SD)	45.0 ± 14.9	15.6 ± 5.6	17.1 ± 5.3	19.4 ± 5.0	20.2 ± 3.9	22.1 ± 4.7
(Minimum - Maximum)	(23.5–78.5)	(6.0–27.5)	(8.0–28.0)	(12.0–31.5)	(14.5–27.0)	(12.0–30.0)
*P*	0.74	0.51	0.85	0.35	0.83	0.53

*Note*: IOPs before surgery and at two weeks, one month, three months, six months and 12 months after surgery were determined using an applanation tonometer (mean of 9 am and 4 pm measurements). Differences in IOPs at various time points throughout follow-up were compared between the two groups using the *t* test

**Table 3:** Success rates of the two groups

	2 weeks	1 month	3 months	6 months	12 months
Injection Group	95.2 %	90.5 %	80.0 %	73.7 %	72.2 %
(Successful subjects/total subjects)	(20/21)	(19/21)	(16/20)	(14/19)	(13/18)
Control Group	90.9 %	81.8 %	77.3 %	71.4 %	68.4 %
(Successful subjects/total subjects)	(20/22)	(18/22)	(17/22)	(15/21)	(13/19)
*P*	0.57	0.41	0.83	0.87	1.00

*Note*: Success was defined as IOP > = 6 mm Hg and < = 21 mm Hg, with or without the use of anti-glaucoma medications, and without severe complications or reoperation. The differences in the success rates were compared between the two groups using the corrected chi-square test (two weeks, one month, and three months) and chi-square test (six months) and two-tailed Fisher’s exact test (12 months)

The two groups showed different patterns of BCVA changes after surgery. In the injection group, BCVA improved significantly and peaked for one to three months before declining sharply to levels comparable to that of the control group. In the control group, BCVA increased slightly, peaked at one month, and then gradually declined. Inter-group statistical analysis showed significant differences only at three-month follow-up (Table 4). BCVAs in both groups fell back to the baseline at six months or 12 months.

**Table 4:** Best corrected visual acuities in both groups

		Pre-surgery	2 weeks	1 month	3 months	6 months	12 months
Injection Group	No.	27.4 ± 20.0	29.3 ± 16.0	39.2 ± 16.6	38.7 ± 16.0	29.7 ± 14.4	24.3 ± 11.1
	LogMAR	1.1 ± 0.4	1.1 ± 0.3	0.9 ± 0.3	0.9 ± 0.3	1.1 ± 0.3	1.2 ± 0.2
Control Group	No.	24.2 ± 19.5	28.7 ± 15.0	30.6 ± 15.6	28.0 ± 14.7	26.4 ± 13.6	22.1 ± 13.9
	LogMAR	1.2 ± 0.4	1.1 ± 0.3	1.1 ± 0.3	1.1 ± 0.3	1.2 ± 0.3	1.2 ± 0.3
*P*		0.65	0.81	0.086	0.045	0.39	0.60

*Note*: Best corrected visual acuities (BCVAs) were determined by ETDRS digital letters before surgery, and two weeks, one month, three months, six months and 12 months after surgery. The differences in BCVAs at various time points throughout follow-up were compared between the two groups using the *t* test

The number of anti-glaucoma medications used at various time points throughout follow-up in both groups declined significantly from baseline (ANOVA, α = 0.05). And the number of medications increased gradually in both groups with the passage of time. There was no significant difference at various time points between the two groups (Table 5).

**Table 5:** Glaucoma medications usage in both groups

	Pre-surgery	2 weeks	1 month	3 months	6 months	12 months
Injection Group	2.5 ± 0.5	0.2 ± 0.4	0.6 ± 0.6	0.7 ± 0.6	1.0 ± 0.7	1.1 ± 0.8
Control Group	2.6 ± 0.5	0.2 ± 0.4	0.7 ± 0.7	0.7 ± 0.7	1.1 ± 0.8	1.3 ± 0.9
*P*	0.66	0.94	0.59	0.93	0.71	0.47

*Note*: The differences in the number of glaucoma medications used at various time points throughout follow-up were compared between the two groups using the *t* test

Of the 21 patients in the injection group, anterior uveitis developed in seven patients one day after surgery, but was resolved with topical steroid drops within two weeks; proliferative membrane formed in the pupil in one patient and was removed using YAG laser. Nine patients experienced mild hyphema, which resolved itself within two weeks. Two patients developed mild vitreous hemorrhage, which resolved itself within a month. Two patients experienced transient ocular hypertension (> = 30 mm Hg) and mild corneal edema one day after surgery due to excessive viscoelastic residues, which were alleviated after eyeball massage. Blood clots developed in the drainage tube in three patients, but resolved themselves in two and were cured by YAG laser at day 3 after surgery. The lens of the eye turned cloudy rapidly in one patient within days after surgery, who underwent cataract surgery two months after surgery and withdrew from this study. Of the 23 patients in the control group, 10 patients developed postoperative anterior uveitis, which was revolved within two weeks after topical steroid drops. Nine patients experienced mild hyphema, which resolved itself within two weeks. Five patients developed mild vitreous hemorrhage, which resolved itself within a month. Four patients experienced transient ocular hypertension (including two combined with corneal edema), which were alleviated after eyeball massage. Blood clots developed in the drainage tube in four patients, but resolved themselves. One patient developed conjunctival retraction at three months which did not cause drainage tube exposure and therefore was left unattended. Mild choroidal hemorrhage in local areas developed in one patient one day after surgery as revealed by B ultrasonography (less than 90°) and resolved itself gradually within two months. No serious complications such as malignant glaucoma, sustained low IOP, endophthalmitis, lose of light perception, and retinal detachment occurred in either of the two groups (Table 6).

**Table 6:** Postoperative complications in both groups

	Injection Group	Control Group	*P*
Anterior uveitis	7 (33.3 %)	10 (47.6 %)	0.42
Worsened corneal edema	2 (9.5 %)	2 (9.1 %)	0.96
Hyphema	9 (42.9 %)	9 (40.9 %)	0.90
Vitreous hemorrhage	2 (9.5 %)	5 (22.7 %)	0.23
Transient ocular hypertension	2 (9.5 %)	4 (18.2 %)	0.41
Malignant glaucoma	0	0	
Sustained low IOP	0	0	
Drainage tube obstruction/displacement/exposure	3 (14.3 %)	4 (18.2 %)	0.73
Lens opacity	1 (4.8 %)	0	
Choroidal hemorrhage/detachment	0	1 (4.5 %)	
Retinal detachment	0	0	
Conjunctival retraction/rupture	0	1 (4.5 %)	
Endophthalmitis	0	0	
Loss of light perception	0	0	
Total	12 (57.1 %)	14 (63.6 %)	0.66

*Note*: The differences in postoperative complications were compared between the two groups using the chi-square or corrected chi-square test

## Discussion

Patients with early NVG have an open angle and normal or slightly elevated IOP. Aggressive treatment with glaucoma medications and laser therapy can bring the disease under control in some of these patients. However, as the disease progresses, the angle is gradually closed and IOP often continues to rise, leading to a poor response to medications or laser, and surgery is usually required. AGV implantation is an effective method for the treatment of NVG, especially for patients with angle closure; however, NVG is associated with unfavorable outcomes [2]. In this study, success was achieved in 71.4 % of the 22 patients in the control group at six months and 68.4 % at 12 months. Some clinical studies using the same surgical procedure have reached similar results. For instance, Yalvac IS et al. [8] performed AGV implantation alone in 38 patients with NVG and achieved success in 63.2 % of the patients at one year. Shen CC et al. [9] reported a success rate of 70 % and 60 % at one year and two years, respectively. In a retrospective study, Netland PA et al. [2] reported a success rate of 73.1 % at one year, 61.9 % at two years, and only 20.6 % at five years, and considered NVG a high risk factor of AGV implantation failure.

The course of NVG depends on the occurrence and development of new vessels in the iris and the anterior chamber angle. Anti-VEGF factors can inhibit intraocular neovascularization, promote atrophy, and mitigate damage to the blood-ocular barrier as a result of leakage from new vessels. A study shows that ranibizumab can lower IOP and alleviate rubeosis in patients with NVG [10]. Therefore, anti-VEGF factors have been used alone or in combination for the treatment of NVG. However, currently available clinical evidence is inconclusive to establish the effectiveness of such drugs, especially in the medium- and long-term [11, 12]. Our study examined the efficacy of AGV implantation with or without a single preoperative injection of 0.5 mg ranibizumab in patients with NVG. After a follow-up period of six months to one year, the results showed that there was no significant difference between the two groups in terms of IOP control, success rate, or anti-glaucoma medications.

Currently available studies on glaucoma treatment have reported the use of anti-VEGF factors under the conjunctiva [13] and in the anterior chamber [7] and vitreous cavity [14–16], and even reported that topical eye drops containing ranibizumab (2 mg/mL) after filtering surgery can reduce the formation of bleb scarring [17]. However, there are large discrepancies in the conclusions reached by a number of small-scale clinical studies on NVG. Elmekawey H et al. [7] injected 0.5 mg ranibizumab into the anterior chamber in 13 patients once and two patients twice, and performed trabeculectomy at four weeks when the new vessels resolved on the surface of the iris. Success was achieved in 93.3 % of the patients at six months. Lüke J et al. [10] used repeated intravitreal injections of ranibizumab for the treatment of iris neovascularization (2.3 times/year) and neovascular glaucoma (3.6 times/year), in combination with traditional therapies such as laser photocoagulation, cryotherapy, and vitrectomy. This treatment approach improved rubeosis and angle closure and achieved effective control of IOP. However, in a retrospective study, Ma KT et al. [18] analyzed the outcomes of NVG patients who received AGV implantation combined intraoperative vitreous injection of 1.25 mg bevacizumab, and found that its one-year success rate did not differ significantly from AGV implantation alone. As our point of view, the possible reasons for the discrepancies of the above studies may include different usages of anti-VEGF factors (especially single or repeated injections) and baseline differences in patients.

Intravitreal injection of anti-VEGF factors can help reduce macular edema and improve vision in patients with RVO and DR [19–21]. Our data showed that early postoperative BCVA improved from baseline in the two groups, because IOP was brought under control and corneal edema was alleviated in most patients. However, with the extension of the follow-up period, BCVA gradually declined, which may be caused by retinal deterioration and worsened cataracts. In comparison, postoperative BCVA improved more notably and this improvement lasted longer in patients administered with ranibizumab (about three months). The average BCVA in the injection group at three months was higher than that in the control group, which is positive for the quality of life and compliance of patients. Nevertheless, the beneficial effect from ranibizumab disappeared thereafter and BCVA started to move closer between the two groups, with no significant difference in medium- and long-term vision outcomes between the two groups. Collectively, our results suggest that single IVR before surgery can only enhance vision in the early period after surgery.

We observed that ranibizumab alleviated rubeosis in patients and this effect started to appear two to three days after administration as measured by slit lamp examination. However, there was no marked difference in the incidence of postoperative complications between the two groups. Early postoperative complications after AGV implantation surgery for NVG included hyphema, choroidal detachment, vitreous hemorrhage, and obstruction of drainage valves. The occurrence of these complications was associated with a number of factors such as the severity of neovascularization of the iris and the anterior chamber angle, the severity of underlying diseases, the level of baseline IOP, and changes in perioperative IOP (especially during surgery). For this reason, we sought to achieve success in our first attempt when preparing a scleral tunnel to avoid sharp IOP decline as a result of repeated puncture of the anterior chamber. Meanwhile an appropriate amount of viscoelastic was injected into the anterior chamber to bring IOP slightly higher than normal levels. The drainage tube was partially ligated using absorbable suture. These measures contribute to the maintenance of anterior chamber and IOP during surgery and within a short period after surgery. Therefore, no serious complications occurred in early postoperative periods in both groups, which, therefore, rendered the role of ranibizumab less significant. Nakatake S et al. [22] studied a group of NVG patients who had received trabeculectomy and also found that the use of bevacizumab injections had no notable effect on the incidence of preoperative complications such as hyphema and choroidal detachment. However, as ranibizumab was applied for a short time, our study failed to establish a correlation between ranibizumab and medium- and long-term complications after AGV implantation, such as drainage valve exposure or fiber encapsulation.

As a non-randomized study, anti-VEGF treatment was assigned at the discretion of the subjects, and the sample size was relative small, so bias would be inevitable between groups. To some extent, our study demonstrates that single IVR before AGV implantation has no significant effect on the medium- and long-term outcomes of NVG patients. As these drugs act in a very time-dependant manner, it is necessary to carry out repeated injections to control the progression of the disease according to changes in rubeosis, IOP, BCVA or the fundus during follow-up. Further studies are needed to explore how to choose and evaluate clinical indicators used to determine the timing of repeated administration. However, we presume that the use of anti-VEGF factors as needed may signal the direction of single or combined treatment modalities for NVG in the future.

## Conclusions

Single intravitreal ranibizumab (0.5 mg) before surgery has no significant effect on the medium- or long-term outcomes of neovascular glaucoma treated with Ahmed glaucoma valve implantation.

## Abbreviations

AGV: Ahmed glaucoma valve
BCVA: best corrected visual acuity
DR: diabetic retinopathy
IOP: intraocular pressure
IVR: intravitreal injection of ranibizumab
NVA: neovascularization of the angle
NVG: neovascular glaucoma
NVI: neovascularization of the iris
PRP: panretinal photocoagulation
RAO: retinal artery occlusion
RVO: retinal vein occlusion
VEGF: vascular endothelial growth factor
